# Feasibility and Effectiveness of a Personalized Home-Based Motor-Cognitive Training Program in Community-Dwelling Older Adults: Protocol for a Pragmatic Pilot Randomized Controlled Trial

**DOI:** 10.2196/49377

**Published:** 2023-11-09

**Authors:** Julia Seinsche, Eling D de Bruin, Enrico Saibene, Francesco Rizzo, Ilaria Carpinella, Maurizio Ferrarin, Sarina Ifanger, Sotiria Moza, Eleftheria Giannouli

**Affiliations:** 1 Motor Control and Learning Group, Institute of Human Movement Sciences and Sport Department of Health Sciences and Technology Eidgenössische Technische Hochschule Zurich Zurich Switzerland; 2 Department of Health OST - Eastern Swiss University of Applied Sciences St Gallen Switzerland; 3 Department of Neurobiology, Care Sciences and Society Karolinska Institutet Huddinge Sweden; 4 Istituto di Ricovero e Cura a Carattere Scientifico Fondazione Don Carlo Gnocchi Onlus Milan Italy; 5 Materia Group Nicosia Cyprus; 6 Division of Sports and Exercise Medicine Department of Sport, Exercise and Health University of Basel Basel Switzerland

**Keywords:** telerehabilitation, home-based, eHealth, exergaming, older adults, motor-cognitive training, feasibility, pragmatic trial

## Abstract

**Background:**

Exergame-based motor-cognitive training in older adults has been associated with improvements in physical, cognitive, and psychological functioning. The novel Cocare system (Dividat GmbH), developed through a user-centered design process, allows motor-cognitive training in a telerehabilitation setting. It includes (1) a stationary stepping platform for supervised exergame training (Dividat Senso; Dividat GmbH), (2) a home-based version (Dividat Senso Flex, which is a rollable pressure-sensitive mat; Dividat GmbH), (3) an assessment system (including motor-cognitive tests), and (4) a rehabilitation cockpit for remote training supervision and management.

**Objective:**

The aim of this study is to test the feasibility and effectiveness of this novel training system.

**Methods:**

A total of 180 older adults from Switzerland, Italy, and Cyprus aged ≥60 years with a prescription for rehabilitation are randomly allocated to an intervention group or a control group. Both groups continue with their usual care, whereas participants in the intervention group additionally perform a 2-week supervised exergame training program at rehabilitation centers, followed by a 10-week home training program under remote supervision. The assessment system is used to indicate the start level of each participant, and, in both intervention periods, standardized progression rules are applied. The measures of feasibility include adherence, attrition, exergame enjoyment, willingness to perform such a training program, and the number and types of help requests. Effectiveness is assessed in terms of cognitive and physical functioning, balance confidence, and quality of life.

**Results:**

Data collection started in February 2023 and is ongoing. Final measurements are expected to be performed in January 2024.

**Conclusions:**

Owing to the user-centered design approach, the Cocare system is expected to be user-friendly and offers several novel features to cover the whole continuum of care. This pragmatic trial will provide valuable information regarding final necessary adaptations and subsequent implementation efforts.

**Trial Registration:**

ClinicalTrials.gov NCT05751551; https://www.clinicaltrials.gov/study/NCT05751551

**International Registered Report Identifier (IRRID):**

DERR1-10.2196/49377

## Introduction

### Background and Rationale

A steep growth in the number of people aged ≥60 years is expected by the end of the 21st century [1]. Age-related declines in physical and cognitive functioning and the associated adverse outcomes, such as restricted mobility, cognitive impairment, and falls, ultimately result in a decrease in the quality of life of older adults. Therefore, the term “active and healthy aging” (AHA) [2] is becoming increasingly important in policy frameworks worldwide.

A common strategy to promote healthy aging is to engage in physical activity. Previous research has shown that physical activity has a number of positive effects on physical and psychological health outcomes as well as on cognition in older adults [3-5]. Furthermore, it has been demonstrated that older adults benefit similarly from cognitive training programs targeting various cognitive functions such as memory, executive functions, attention, visuospatial skills, and information processing speed [6].

Given the comprehensive positive effects of physical activity and cognitive training, it has been suggested that a combination of both might be promising. Indeed, previous studies have revealed that simultaneous motor and cognitive training may be equally effective as, or even more effective than, separate or sequential training of both functions [7-13]. In fact, simultaneous motor-cognitive training can lead to improvements in physical functions such as balance [14-16], aspects of gait (eg, gait initiation) [17], and movement quality [18] as well as in cognitive functions such as executive control and processing speed [19,20], exercise enjoyment [21], decreased depressive symptoms, and an increased mental health–related quality of life [22].

However, several issues are limiting the success of such training programs; for instance, because most physical and cognitive training programs are provided face-to-face, accessibility is a major concern for older adults owing to potential mobility limitations, transportation issues, or a lack of facilities in their nearby community. Furthermore, demographic changes are increasing the need for long-term care and treatment [23] and pose financial, time, and personnel challenges to health care systems. As a result, training interventions can often not be provided for long enough durations, thus preventing patients classified as geriatric from reaching their full recovery potential and from leading an active and healthy lifestyle [24].

A solution to the aforementioned obstacles is offered by information and communication technologies, which can support the provision of care through telerehabilitation. Telerehabilitation is a broad term and can be defined as the remote provision of rehabilitation services [25]. Such a transfer of postacute rehabilitation to the home setting potentially offers a cost-effective solution to meet the increased demands on health care services, may solve the problem of accessibility, and can therefore play a key role in AHA. In addition, telerehabilitation is especially important during lockdown periods, such as those experienced during the recent COVID-19 pandemic.

Exergames are a promising option to provide telerehabilitation by means of simultaneous motor-cognitive training. They can be described as interactive whole-body movement game-based training approaches that efficiently connect motor and cognitive tasks [10]. This is the basic idea of the Cocare system (Dividat GmbH), a novel multicomponent exergame-based telerehabilitation system, including a stationary (Dividat Senso; Dividat GmbH) as well as a portable (Dividat Senso Flex; Dividat GmbH) exergame hardware device, an assessment system, and a digital training management system—the so-called rehabilitation cockpit. A more detailed description of the system can be found in the study by Seinsche et al [26].

Interventions with the Cocare system can be defined as complex interventions considering *“*the number of components involved; the range of behaviors targeted;...the number of groups, settings, or levels targeted; or the permitted level of flexibility of the intervention or its components*”* [27]. Implementation-based complex intervention research is primarily focusing on how to develop interventions that are implementable, cost-effective, and transferable to real-world settings and across different contexts [27,28]. Thereby, complex intervention research comprises four phases: (1) development of the intervention, (2) testing its feasibility, (3) evaluating the intervention, and (4) implementation of the intervention. In each phase, a major core element is the engagement of appropriate stakeholders, including those who will deliver and use the intervention [27,28]. Following this framework for the design and evaluation of complex interventions, the Cocare project was initiated to develop and validate the Cocare system applying an iterative user-centered design process to constantly refine the components of, and interventions with, the Cocare system. Previously, a focus group study was conducted to identify primary (older adults) and secondary (health care professionals) end-users’ needs and requirements regarding an exergame-based telerehabilitation system in general as well as the Cocare system specifically [26]. On the basis of the results, the Cocare system was adapted before being tested in a usability study that examined the perceived usefulness as well as the ease of use, acceptability, and enjoyment of the training approach [29]. In this usability study, the Cocare system received good acceptance by primary and secondary end users, with the primary end users indicating high enjoyment while playing the exergames. However, barriers such as difficulties in understanding certain assessment and game instructions were mentioned as well. After repeated adjustments, phases 2 and 3 of complex intervention research are now targeted (ie, testing the system’s feasibility and conducting a preliminary evaluation of its effectiveness).

### Objectives

The objectives of this study are (1) to test the feasibility of a personalized exergame-based motor-cognitive telerehabilitation training approach using the Cocare system in older adults with a prescription for rehabilitation (inpatient or outpatient) and (2) to obtain first insights into the effects of this training approach on cognitive and motor functions, balance confidence, and quality of life.

## Methods

### Trial Design

To be useful for decision makers, complex intervention research should take into consideration the complexity based on the intervention’s components as well as on its interaction with the context in which it is being implemented [27], which is why a pragmatic design approach was chosen. Thus, this study is conducted as an international pragmatic pilot randomized controlled trial (RCT) with 2 arms (parallel groups). The SPIRIT (Standard Protocol Items: Recommendations for Interventional Trials) 2013 checklist was used for the reporting of this pragmatic RCT [30]; however, the item order has been modified slightly for good comprehensibility of our study procedures.

### Ethical Considerations

The study protocol was approved by all local ethical committees, including the Cantonal ethics committee in Zurich, Switzerland (2022-01746); the Don Carlo Gnocchi Foundation ethics committee in Italy (06_16/12/2022); and the Cyprus National Bioethics Committee (ΕΕΒΚ ΕΠ 2021 51). It has been registered at ClinicalTrials.gov (NCT05751551). Any amendments of the protocol are noted, and substantial changes will be reported to the ethics committees. All interested participants receive an information sheet and a consent form describing the study and providing sufficient information for the participant to make a decision regarding study participation. A team member (ie, the principal investigator or other investigators involved) arranges a personal meeting with the prospective participants and informs them again about all study procedures, benefits, risks, and their rights regarding study participation. Afterward, the consent form is signed by both—the participant and the investigator—before the participant undertakes any study procedure. The informed consent process is documented in REDCap (Research Electronic Data Capture; Vanderbilt University).

### Participants

#### Study Setting

This study is an international multicenter RCT, involving an intervention group (IG) and a control group (CG), conducted in three countries or trial sites: (1) Switzerland (ETH Zurich in collaboration with VAMED Rehabilitation Center Zurich Seefeld, Zurich), (2) Italy (Don Carlo Gnocchi Foundation, Milan), and (3) Cyprus (Materia Group, Nicosia). Each trial site follows detailed standard operating procedures that have been developed before the study start for each step of the study, including recruitment, measurements, intervention, and data management.

#### Eligibility Criteria

Interested participants are screened for eligibility via telephone and in person. The inclusion criteria are (1) aged ≥60 years (2) a prescription for rehabilitation (as inpatient or outpatient) within the past 6 months, (3) Mini-Mental State Examination (MMSE) score ≥24, (4) physically able to independently stand for at least 2 minutes, (5) able to give informed consent, (6) internet access at home, and (7) availability of a television or a large screen at home.

The exclusion criteria are (1) permanently living in a nursing home, (2) mobility limitations or comorbidities impairing the ability to perform a step-based motor-cognitive training program, (3) cognitive limitations or comorbidities impairing the ability to perform a step-based motor-cognitive training program, (4) severe sensory impairments, (5) previous or acute major psychiatric illness (eg, schizophrenia, bipolar disorder, and recurrent major depression episodes), (6) a history of drug or alcohol abuse, (7) terminal illness, (8) participation in another clinical trial, and (9) absence from home of >2 weeks during the study period.

### Intervention

#### Overview

The study comprises two intervention periods: (1) a 2-week supervised exergame-based intervention period at the rehabilitation center and (2) a home-based exercise intervention period of 10 weeks (a total of 12 wk considering a maximum permitted interruption of 2 wk). The first period serves as the supervised familiarization of the participants with the training approach before continuing training in their homes without direct supervision. The second period starts with the study investigators visiting the participants at home to provide guidance and safety instructions for the training program and to set up the system. After 5 weeks of home-based training, the study investigators visit IG participants again for major adaptations in the training plan. For the second half of this intervention period (week 6-week 10), a new selection of games is implemented to create sufficient variety and thus minimize boredom. In addition, participants in both groups receive biweekly telephone calls by a study investigator to collect data about their usual care as well as daily physical and cognitive activities and to ask about the perceived level of training difficulty. Adherence to, and performance in, the training sessions are supervised remotely using the rehabilitation cockpit, which also serves for remote training management or progression setup.

#### Description of the Training Device and the Motor-Cognitive Intervention

The device that delivers the exergame training at the clinic is the Dividat Senso, which is a stepping platform with 5 pressure-sensitive plates connected to a screen on which the stimuli of the exergames appear. The exergames are played by taking steps in 4 directions (front, back, left, and right) or body weight shifting. This approach has been shown to be feasible for motor-cognitive training in older adults and has proven effective in improving various motor and cognitive outcomes [14,31-33]. For the home-based training program, Dividat Senso Flex (a portable version of the Dividat Senso hardware) is used. This is a foldable pressure-sensitive mat that is also connected to a television or screen. The Senso Flex system allows the same functionalities as the stationary Senso system. The motor-cognitive training with the game software targets different cognitive functions, such as attention, executive functions, and memory, and visuospatial functions as well as balance and strength.

Before starting the first intervention period, a series of physical and cognitive assessments are conducted to evaluate the participants’ functional status and to create individual training plans. In both intervention periods, participants are instructed to train 3 times per week for 20 minutes, but they are allowed to divide total training volume (3 × 20 = 60 min) into more training sessions or to merge training sessions (eg, 2 × 30 = 60 min) if they wish. A training plan consisting of 15 levels of difficulty for each trained function (cognition, balance, and endurance) has been created with the levels progressing in terms of (1) choice of more difficult games and (2) increasing game duration. The initial training level is determined based on each participant’s functional status.

Subsequently, training progression and personalization are carried out based on three factors: (1) a progression algorithm integrated into the software adapting the level of difficulty in each game in real time, (2) an objective progression criterion (performance plateau, defined as <5% performance increase from 1 week to the other [34]), and (3) a subjective rating of the level of difficulty by the participants based on the Borg Ratings of Perceived Exertion Scale [35] indicating whether a game was too exhausting or too easy. If, in the context of progression, new games are implemented in the training plan, the participants are informed accordingly by written messages sent via the rehabilitation cockpit.

With our training plan, we aim to follow the findings of previous systematic reviews that suggest that to be effective, a combined motor-cognitive training approach should be applied for a minimum of 8 weeks, 2 to 4 times a week, and should be based on tailored intensity monitoring and progression [10,11,36].

#### Usual Care and Choice of Comparator

Both IG and CG participants continue their usual care throughout the study. In pragmatic trials, usual care is difficult to define and could theoretically include a large range of interventions [37]. It could be argued that CG participants should receive 1 specific predefined treatment, but “patients in usual care control group should be confronted with the heterogeneity of treatments available in real, daily practice, rather than receiving a treatment chosen by the researchers” [37]. For this reason, we aim to administer the content of the usual care programs as precisely as possible through training logs.

#### Criteria for Discontinuing or Modifying Allocated Interventions

The criteria for which a participant is withdrawn from the study are (1) the withdrawal of informed consent, (2) acute hospitalization, (3) the occurrence of events or diseases listed in the exclusion criteria, and (4) the occurrence of any intervention-related major safety issues. In case of the last-named criterion, the study will be discontinued.

#### Strategies to Improve Adherence to Interventions

During the home-based training period, regular telephone calls (4 times) and personal visits (2 times) are made, which are expected to improve the participants’ adherence. In addition, written motivational and feedback messages can be sent to the participants via the rehabilitation cockpit.

#### Relevant Concomitant Care Permitted or Prohibited During the Trial

There is no relevant concomitant care or intervention that is specifically permitted or prohibited during the intervention.

### Outcomes and Outcome Measures

#### Primary Outcome

The primary outcome of this study is the feasibility of a home-based exergame training program with the Senso Flex in older adults. Thus, we follow the phases defined in the updated framework for developing and evaluating complex interventions commissioned jointly by the Medical Research Council and the National Institute for Health Research and recommended for the development of complex interventions [27]. For this purpose, we assess adherence, attrition, and perceived enjoyment in IG participants (Table 1).

The adherence rate is determined by calculating the duration of completed training sessions as percentages of the recommended duration of training. Furthermore, the attrition rate in both groups is calculated as the percentage of dropouts during study participation.

Perceived enjoyment while playing the exergames is measured with the Exergame Enjoyment Questionnaire, developed to assess enjoyment of the physical activity aspect in exergames together with enjoyment of the gaming elements [38]. The questionnaire comprises 20 items that are rated on a 5-point Likert scale, resulting in a minimum possible score of 20 and maximum possible score of 100. Considering the results of previous literature [38] and of a preliminary study testing the usability of this motor-cognitive training approach [29], an average Exergame Enjoyment Questionnaire score of 70 points (out of 100) can be deemed as *acceptable*.

However, the criteria for trial success are solely based on adherence and attrition rate measures. A 70% adherence rate was defined as being “adherent to the training program” [46,47]. According to a systematic review [47], an attrition rate of ≤10% is *acceptable* for RCTs. However, because this study is a pragmatic RCT, we deem an attrition rate of ≤20% as *acceptable*.

**Table 1:** Outcomes, outcome measures, and time points.

Category and outcome			Assessment	Assessment time point
**Primary outcomes**				
	**Feasibility**			
		Adherence	Adherence rate	IP 2^a^
		Perceived enjoyment	EEQ^b^ [38]	T3^c^
		Attrition	Number of dropouts	Whole study period
**Secondary outcomes**				
	**Feasibility**			
		Subjective workload	NASA-TLX^d^ [39]	T3
		Willingness to continue home-based exergame training	Single question	T3
	**Cognition**			
		Psychomotor speed	Reaction time test	T2^e^ and T3
		Selective attention	Go-no-go test [40]	T2 and T3
		Cognitive flexibility	Flexibility test [40]	T2 and T3
		Information processing	Flexibility test [40]	T2 and T3
	**Balance**			
		Static balance	Tandem Romberg test [41]	T2 and T3
		Dynamic balance	Coordinated stability test	T2 and T3
		Functional balance	Timed up-and-go dual task [42]	T2 and T3
		Dual task	Timed up-and-go dual task [42]	T2 and T3
	**Strength**			
		Mobility	Timed up-and-go dual task [42]	T2 and T3
		Leg power	30-s chair-stand test [43]	T2 and T3
	**Psychological factors**			
		Balance confidence	ABC^f^ Scale [44]	T2 and T3
		Quality of life and satisfaction with health	Two questions	T2 and T3
**Other outcomes**				
	**Usual care**			
		Usual care and daily physical and cognitive activities	Self-made questionnaire	Whole study period
	**Training**			
		Training time (IG^g^)	—^h^	IP 2
	**Demographics**			
		Age, sex, years of education, living situation, body weight and height, reason for therapy prescription, and number of falls	Questionnaire	T1^i^
		Comorbidities	Charlson Comorbidity Index [45]	T1

^a^IP 2: intervention period 2 (home-based exercise intervention period of 10 wk).

^b^EEQ: Exergame Enjoyment Questionnaire.

^c^T3: after the intervention.

^d^NASA-TLX: National Aeronautics and Space Administration Task Load Index.

^e^T2: before IP 2.

^f^ABC: Activities-Specific Balance Confidence.

^g^IG: intervention group.

^h^Training time is automatically recorded by the system and presented in the rehabilitation cockpit.

^i^T1: at study entry.

#### Secondary Outcomes

##### Overview

Several other feasibility measures are assessed as secondary outcomes (Table 1). The National Aeronautics and Space Administration Task Load Index [39] is used to assess the subjective workload during exergaming. It consists of 6 subscales: mental demand, physical demand, temporal demand, performance, effort, and frustration. Each subscale is rated on a 20-point Likert scale ranging from 0=very low to 20=very high. The raw National Aeronautics and Space Administration Task Load Index scores are calculated by multiplying each subscore by 5.

In addition, after the intervention, participants will be asked about their willingness to continue a similar home-based exergame training program after the end of the study (as a proxy for training satisfaction), and agreement will be determined on a 5-point Likert scale. Finally, during the whole home-based intervention period, the number and types of additional instructions as well as help instructions are noted.

Furthermore, a series of physical and cognitive functions as well as balance confidence are assessed before and after the home-based intervention as secondary outcomes to obtain first indications regarding the effectiveness of the home-based exergame intervention (Table 1).

##### Cognitive Assessments

The cognitive assessments are mainly based on the standardized and validated *Test for Attentional Performance* (TAP) developed by Zimmermann and Fimm [40], a collection of reaction time tasks with tasks consisting of “simple and easily distinguishable stimuli that the patients react to by a simple motor response*”* [48], and are conducted using the Dividat Senso.

**Figure 1:**
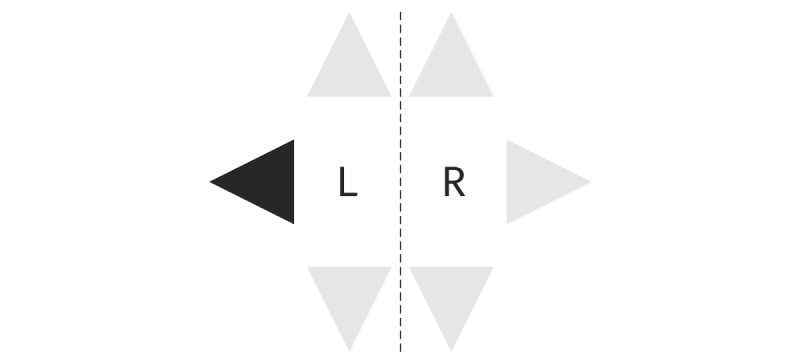
Reaction time test.

The reaction time test measures psychomotor speed. Six triangles are presented on the display. When a triangle turns dark, the participants are asked to react as fast as possible by taking a step in the corresponding direction (Figure 1). The arrows also indicate with which foot the step must be taken. The average reaction time (ms) and errors will be included in the analysis of effectiveness.

**Figure 2:**
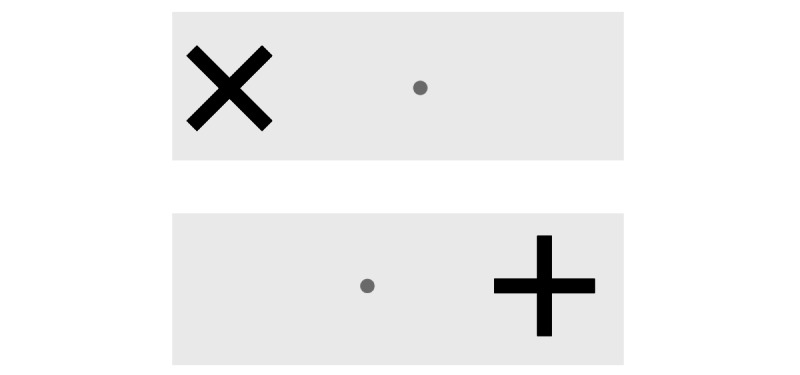
Go-no-go test.

The go-no-go test was developed based on the go-no-go test from the TAP assessment battery [40] and measures selective attention and inhibition. More precisely, it evaluates the ability to suppress a response in the presence of irrelevant stimuli as well as the response latency during stimulus selection. Participants are asked to react as fast as possible when a cross (x) is presented on the screen. However, in case the stimulus is a plus sign (+), they must remain still (Figure 2). The average reaction time (ms) will be included in the analysis of effectiveness, and errors will be described descriptively.

**Figure 3:**
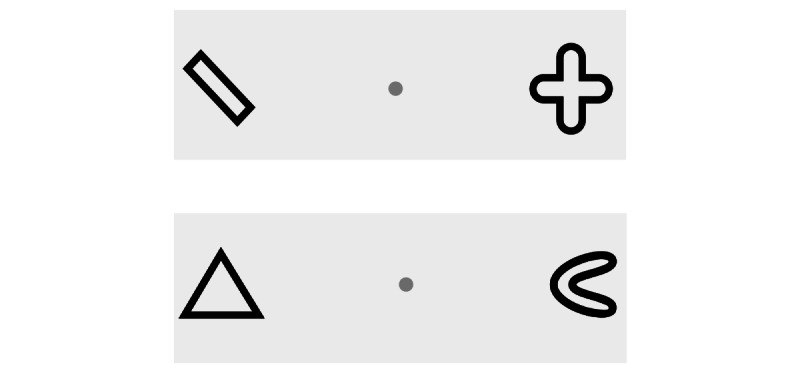
Flexibility test.

The flexibility test is based on the flexibility test from the TAP battery [40] and aims to assess the flexibility of focused attention. Participants are instructed to react by taking a step toward the rounded figure and then toward the angular figure in an alternating manner (Figure 3). The average reaction time (ms) and errors will be included in the analysis of effectiveness.

##### Balance

The sway test is based on the Romberg test [49,50] and is conducted on the Senso. The participants are instructed to stand as still as possible for 30 seconds. Meanwhile, postural sway, which is the movement of the center of pressure, will be recorded. The sway path length (mm) will be used for further analyses.

The coordinated stability test serves to measure dynamic balance. At the beginning, the participants are instructed to stand with their feet hip width apart on the middle plate and keep their arms crossed in front of their chest for the entire duration of the assessment. The task is then to follow a figure shown on the screen by shifting their center of pressure without lifting their feet. The output metrics, which will be included in the analysis of effectiveness, are the difference from the ideal path (mm) and the completeness of the path (percentage).

The timed up-and-go test [42] is a measure of mobility and functional balance and requires the participant to *“*rise from a standard armchair, walk to a line on the floor 3 meters away, turn, return, and sit down again*”* [42]. The timed up-and-go dual task (DT) complements this motor task (as the primary task) with a secondary task—here a cognitive task. For the cognitive task, participants are instructed to count backward from 90, subtracting 7 successively. This simultaneous execution of motor and cognitive tasks reflects real-life conditions better, considering the fact that in older adults, gait is not only a motor activity but also involves cognitive demands [51]. Two attempts without DT and 2 attempts with cognitive DT are conducted. In both cases, the time until the patient is fully seated again is recorded, and the best attempts are used for further analysis. DT cost is calculated by determining the percentage at which the secondary task interfered with the test performance:

DTC [%] = 100 × (DT score − simple task score) / simple task score **(1)**

where “DTC” is the DT cost [52].

##### Leg Power

The 30-second chair-stand test [43] is used to assess lower limb power and short-term endurance. The participants start seated on a chair; after a countdown, they must stand up straight and sit down again as many times as possible within a time frame of 30 seconds. Meanwhile, the number of times the patient fully stands up from a seated position is counted.

##### Balance Confidence

The Activities-Specific Balance Confidence Scale measures the fall-associated self-efficacy of a person in different activities and situations (eg, stair climbing and walking on icy pavements). The scale is able to detect the loss of balance confidence in highly functioning older adults too [44].

##### Quality of Life

Participants are asked to rate their overall quality of life and satisfaction with their health. Both assessments use a 5-point Likert scale (ranging from 1=very poor or very dissatisfied to 5=very good or very satisfied).

##### Other Outcomes

In addition, every 2 weeks, all participants are queried by a study investigator regarding the dose (frequency and time) and content (intensity and type) of their usual care as well as their daily cognitive and physical activities, and the training times of the IG participants are collected.

At baseline, the following demographic information is collected to describe the study population: age, sex, years of education, living situation (living with another person or alone), body weight and height, reason for therapy prescription, number of falls, and comorbidities (Charlson Comorbidity Index) [45].

### Assessment Time Points and Duration

Assessments are performed in a standardized order at study entry (T1), before intervention period 2 (T2), and after the intervention (T3; Tables 1 and 2). Depending on group allocation, the T1 assessment takes 30 to 60 minutes and mainly serves to collect demographic data, randomize participants, and, in the case of IG participants, assess the functional status to subsequently create their training plan. The T2 and T3 assessments represent pre- and postintervention measurements for the evaluation of the home-based intervention’s effectiveness. Both measurements take approximately 90 minutes to complete.

**Table 2:** Schedule of interventions and assessments.

Study phase	Information and screening; phone call and study center^a^	Inclusion and baseline (T1); study center^a^	Intervention period 1; study center	Measurements before intervention period 2 (T2); study center	Intervention period 2; home setting^a^	Postintervention measurements (T3); study center
Oral and written information	✓	✓				
Eligibility criteria	✓	✓				
Written consent		✓				
Baseline factors		✓				
Primary outcomes					✓ (IG^b^ only)	✓ (IG only)
Secondary outcomes				✓	✓ (IG only)	✓
Intervention			✓ (IG only)		✓ (IG only)	
Other outcomes: training time			✓ (IG only)		✓ (IG only)	
Other outcomes: usual care			✓		✓	

^a^Mode and location.

^b^IG: intervention group.

### Participant Timeline

#### Overview

Table 2 displays the time schedule of enrollment, interventions, and measurements for IG and CG participants, as well as the respective data collected at each time point.

### Sample Size

An intended sample size of 180 is based on recommendations from Whitehead et al [53], who stated that, in pilot clinical trials, for an extra small standardized effect size (≤0.1) and 90% power, 75 participants per group are required. We are expecting a 20% dropout rate, which is why we aim to recruit 90 participants per group; thus, in total, 180 participants (n=60, 33% at each trial site).

### Recruitment

In Switzerland, participants are recruited through VAMED Rehabilitation Centre Zürich Seefeld, through an advertisement in the University of the Third Age of the University of Zurich, through announcements to various senior centers and organizations, and through university newsletters. Furthermore, participants of previous studies on older adults are contacted. Participants in Italy are recruited via convenience sampling, and in Cyprus, older adults who are members of existing networks are contacted, and collaborations with multifunctional centers for older adults are used. Besides, an open invitation to the general public has been published.

### Assignment of Interventions

#### Sequence Generation, Allocation Concealment, and Implementation

Participants are randomly allocated to either the IG or the CG with a 1:1 allocation ratio using permuted block randomization to avoid group size imbalances as well as allocation predictability. Blocks of 4, 6, and 8 were randomly generated. The allocation list was created with the web-based randomization tool Sealed Envelope [54] and uploaded on REDCap by a study investigator not involved in the enrollment, assessment, and randomization of participants. Randomization is then run in REDCap after baseline assessments. This allows a careful allocation concealment, and the investigators responsible for recruitment and baseline assessments are unaware of the group to which a participant will be allocated.

#### Blinding

Given the nature of a pragmatic RCT, neither the investigators nor the participants are blinded. The investigators will be responsible for supervising the interventions as well as for conducting the measurements, and participants cannot be blinded because the CG participants do not receive any intervention (other than usual care).

### Data Management and Analysis

#### Data Management

All investigators received theoretical and practical trainings about all study procedures, including data collection and entry. Besides, written instructions have been created to ensure standardized conduction and reproducibility of the study protocol. These trainings were documented noting date, training time point, trainer name, trainee names, and training content.

All data of all study participants are kept strictly confidential. Data are coded, and the key remains password protected in a secure network folder.

REDCap, an electronic data capture system that meets the requirements of Good Clinical Practice, is used for electronic data entry. To ensure data quality, ranges for data values have been prespecified in REDCap, which will give a warning sign if the entered data lie outside these ranges. In addition, all data entered are double-checked by an investigator not involved in specific data collection who then confirms data accuracy with their signature.

#### Statistical Analysis

Baseline data of IG and CG participants will be compared with independent, 2-tailed *t* tests to validate the comparability of the 2 groups. The feasibility measures will be analyzed in a descriptive way. Besides, qualitative reports will be created based on the notes of the investigators regarding the kind of help requests and additional instructions, as well as other observations.

A generalized linear mixed model will be built to analyze changes in the measures of effectiveness for the IG participants compared with the CG participants (time × group interaction to indicate whether change differs between the groups). Thus, the explanatory variables include time (T2 and T3), group (IG or CG), and the interaction between time and group. To estimate the size and direction of effects, effect sizes (Pearson correlation coefficient *r*) and CIs will be calculated [55].

Per-protocol analysis will be conducted including data of only those participants who underwent the whole study procedure, with a training adherence of at least 70% of the training at home.

In case of missing data, a missing value imputation method will be used.

### Monitoring

#### Overview

An internal monitoring visit was conducted at ETH Zurich before the inclusion of the first participants. Further monitoring visits will be conducted after the inclusion of the first participants, throughout the whole study period to track the study progress, and at closeout, which will ensure data quality and the early detection of possible mistakes. Moreover, the clinical trial unit of the Don Carlo Gnocchi Foundation as well as the chief scientist of Materia are monitoring this study. Furthermore, there are regular meetings with the whole study team for the principal investigator to check on and manage the accuracy of the study procedures at all trial sites.

#### Adverse Event Reporting and Harms

In case of an adverse event, we will investigate whether it was caused by the study intervention. Furthermore, a severity assessment of the event as mild, moderate, or severe will be made. If necessary, a report will be sent to the local ethics committees.

#### Auditing

The ethics committees may conduct a quality assurance audit of this study.

### Dissemination Plan

The study results will be disseminated to academic groups and the public through publications, conference presentations, and social media.

## Results

Data collection started in February 2023 and is still ongoing. As of May 2023, we enrolled 77 participants. Final measurements are expected to be performed in January 2024, and results are expected to be published in spring 2024.

## Discussion

### Study Rationale

This pragmatic RCT will provide new knowledge on the feasibility and effectiveness of an exergame-based telerehabilitation program in older adults.

Despite the promising results of previous studies investigating telerehabilitation and exergames in older adults, the feasibility and effectiveness of a combination of both have not yet been sufficiently demonstrated. Furthermore, the combined provision of both approaches (motor-cognitive training) performed in a home setting and independently by older adults is another novel, underexplored, and fertile research area tapped by this study. To the best of our knowledge, previous research has either investigated telerehabilitation approaches that rely on the consecutive conduction of motor and cognitive training [56], or, in the case of approaches that rely on exergames, these have often limited their focus to specific populations (eg, survivors of stroke [57-60]; patients with Parkinson disease [61,62]; and older adults with cognitive impairment [63], subsyndromal depression [22], or lower limb amputation [64]) or healthy older adults [65-67]. However, exergame-based telerehabilitation should be feasible and effective in a broader population of both healthy and recovering older adults. It should especially target those without long-term access to traditional rehabilitation interventions and should accompany them to full recovery.

For this reason, and given the nature of a pragmatic RCT, in this study, the inclusion and exclusion criteria are formulated in a rather broad way to analyze the feasibility of a motor-cognitive telerehabilitation intervention in a diverse population of older adults to mirror or reflect real-life conditions and usual clinical practice as best as possible. Therefore, only participants who are clearly unable to perform a step-based motor-cognitive training program owing to physical and cognitive limitations must be excluded (another reason for exclusion: they cannot be regarded as future targets of such intervention approaches).

Concerning the study design, pragmatic RCTs have a limited internal validity, but they provide real-world evidence as well as generalizability to many real-world settings and therefore high external validity. Thus, they are “becoming a major tool in the evaluation of complex interventions and services” [68].

### Conclusions

The Cocare system is complex and innovative in terms of 4 aspects. First, it includes an assessment system comprising cognitive as well as physical assessments that are based on the validated TAP battery [40]. Second, it introduces in a home environment an exergame-based training approach that has previously been proven to be effective in inpatient rehabilitation of patients classified as geriatric [31]. This seems promising, given decreasing access to conventional supervised interventions owing to the demographic change. Third, the training program can constantly be monitored and managed by health care professionals who can also communicate with the participants directly through the system (eg, send them motivational or feedback messages). Finally, and most importantly, the system has been developed in an iterative user-centered design process, which previous system developers have failed to do [69,70]. Thus, after being adapted based on a focus group study and a usability study, the Cocare system is ready to enter phases 2 and 3 of complex intervention research, and an acceptable feasibility and effectiveness can be expected.

## Abbreviations

AHA: active and healthy aging
CG: control group
DT: dual task
IG: intervention group
MMSE: Mini-Mental State Examination
RCT: randomized controlled trial
REDCap: Research Electronic Data Capture
SPIRIT: Standard Protocol Items: Recommendations for Interventional Trials
TAP: Test for Attentional Performance

## Data Availability

Pending approval by the responsible local ethics committees, all data will be publicly available on Zenodo. Otherwise, data will be available to researchers upon reasonable request.
